# The PIC Cystogram: Its Place in the Treatment Algorithm of Recurrent Febrile UTIs

**DOI:** 10.1155/2008/763620

**Published:** 2008-07-29

**Authors:** Jennifer A. Hagerty, Max Maizels, Earl Y. Cheng

**Affiliations:** Department of Pediatric Urology, Children's Memorial Hospital, 2300 Children's Plaza, Box 24, Chicago, IL 60614, USA

## Abstract

*Purpose*. A common pediatric dilemma involves management of children with recurrent febrile urinary tract infections (UTIs) who have normal voiding cystourethrograms. Vesicoureteral reflux (VUR) has been demonstrated in such cases by performing a cystogram which positions the instillation of contrast (PIC) at the ureteral orifice. We describe the evidence supporting this diagnostic test. *Materials and Methods*. The literature was searched to identify and subsequently evaluate all studies investigating PIC cystography. *Results*. In patients with febrile UTIs and negative VCUGs, the PIC cystogram has been demonstrated to identify occult reflux (PIC-VUR). When identified and treated, these patients have a significant reduction in the incidence of febrile UTIs. *Conclusions*. Although the current literature on PIC cystography is limited, it appears to be a clinically useful test in a select group of patients with recurrent febrile UTIs, that are not found to have VUR on a conventional VCUG. A prospective randomized trial is underway to further define its role in the treatment algorithm of febrile UTIs.

## 1. INTRODUCTION

UTIs result in over 1 million physician visits annually, affecting from 2.4% to 2.8% of children. Reference [1] Many of these patients will go on to have recurrent infections. Reference [1] Recurrent febrile UTIs, while not a proven risk for renal damage, contribute to economic burdens for the family and society because of the recurrent medical needs and hospitalizations. Inpatient hospital costs alone are estimated to be greater than 180 million dollars per year [1]. These figures do not consider the societal impact of such UTIs on days children lose from school or parents miss work.

Less than 50% of patients with febrile UTIs demonstrate VUR. Despite an adequate work up to include characterization of the type and source of bacteria, upper tract evaluation to include renal ultrasound and DMSA renogram, and lower tract evaluation to include voiding cystography and diagnosis of dysfunctional elimination syndrome (DES), the etiology of recurrent febrile UTIs often remains elusive. The empiric management of these patients often involves administering antimicrobials intermittently when infections occur or chronically as prophylaxis.

Because we have been dissatisfied with such empiric management, we have pursued further testing for reflux in such patients with recurrent febrile UTIs who show no evidence of reflux on a conventional VCUG. This testing is known as the positioned instillation of contrast (PIC) cystogram. This is done during cystoscopy by positioning the instillation of contrast at the ureteral orifice under fluoroscopic control. This is a test to check for VUR that may be clinically significant yet was not identified on the conventional VCUG. The historical evolution of this test was based upon observations that many of these children were found to have patulous orifices that easily distended with the flow of water (i.e., hydrodistention) when they were evaluated endoscopically. Noted hydrodistention was then followed by checking for VUR using radiographic contrast fluid. As the reflux was demonstrated on PIC, but not by conventional VCUG, it is termed occult. We detail the current knowledge regarding the test and our view on its place in the current management scheme of children with recurrent febrile UTIs who do not have VUR on conventional VCUG.

## 2. PIC CYSTOGRAPHY TECHNIQUE

PIC cystography is performed at the time of cystoscopy. After induction of general anesthesia, the child is placed in the dorsal lithotomy position. Using a rigid cystoscope, the urethra and bladder are systemically evaluated for anatomical abnormalities such as ureteroceles, diverticuli, and mucosal abnormalities. The ureteral orifices are identified and evaluated for their position and trigonal appearance. The ureteral orifices are then evaluated for insufficiency, VUR, as follows:

- the bladder is emptied,
- the cystoscope beak is positioned facing the ureteral orifice, close enough to the ureteral orifice so that the orifice fills the cystoscopic view but not inside the orifice,
- contrast to be instilled is placed at a height of 1 meter above the level of the bladder. This is the height of contrast flow done for a conventional VCUG,
- via the irrigation port of the cystoscope, contrast is flowed toward the ureteral orifice while fluoroscopy is done,
- the bladder is then emptied and the procedure is repeated on the contralateral side [2].

If the ureteral orifice is insufficient to prevent reflux of contrast, hydrodistention will be noted at cystoscopy and VUR will be imaged by fluoroscopy.

## 3. PILOT STUDIES ON PIC CYSTOGRAPHY

In 2003, Rubenstein et al. introduced the technique and their experience in using this test. Fifty seven children who underwent cystoscopy were evaluated. The data was analyzed by comparing the results in a control group versus those in the study group. The control group was comprised of 2 sets of patients: (a) patients not expected to demonstrate VUR as there was not a history of febrile UTI, the ultrasound was normal, and the conventional VCUG was normal (15 patients, 30 ureteral orifices) and (b) patients expected to demonstrate VUR on a PIC cystogram as there was a history of febrile UTI and VUR was seen on conventional VCUG (12 patients, 24 ureteral orifices). The study group was comprised of patients with recurrent febrile UTIs, a normal ultrasound, and a normal VCUG (30 patients, 60 orifices) [2]. The analysis of data for the control group (a) in which all patients were not expected to demonstrate VUR demonstrated that all orifices appeared normal and none demonstrated PIC-VUR. In the analysis of data for the control group (b) in which all patients had VUR on conventional VCUG, all 15 ureteral orifices with known VUR showed lateral ectopia and/or patulous morphology, and hydrodistention and also demonstrated PIC-VUR. A total of four ureteral orifices appeared normal and did not exhibit VUR by VCUG or PIC cystography, and 5 were lateral and/or patulous and did not demonstrate VUR by VCUG but did show PIC-VUR. From this data, Rubenstein concluded that PIC cystography was 100% sensitive and 91% specific in identifying VUR [2]. These findings in the control group are very important since this demonstrates that a patient with a normal orifice will not artifactually reflux with PIC cystography.

In the study group, all 30 patients had at least one orifice with abnormal morphology. PIC-VUR was identified in all these patients with a history of febrile UTIs. All were treated for VUR with either antimicrobial prophylaxis or reimplantation. During 8-month followup, no patients experienced a recurrent febrile UTI [2].

More recently, Tareen et al. performed a similar study in a small number of patients resulting in their recommendation that the PIC cystogram should be part of the algorithm in evaluating patients with recurrent febrile UTIs without VUR on VCUG. All 5 patients in this study with radiographic confirmation of pyelonephritis showed PIC-VUR. All were treated with endoscopic injection of dextranomer/hyaluronic acid copolymer or vesicoureteral reimplantation. In a followup from 11 to 16 months, no patient has had recurrence of febrile UTIs [3].

From these initial reports, it is concluded that occult VUR identified by PIC cystography may provide an explanation for recurrent febrile UTIs in patients with otherwise negative radiographic studies.

## 4. MULTI-INSTITUTIONAL EVALUATION OF PIC CYSTOGRAPHY

These initial experiences with treatment of VUR demonstrated by PIC cystography for febrile UTIs sparked the establishment of a multi-institutional registration of cases by Edmondson et al. [4]. Four centers performed PIC cystography on 39 consecutive patients with febrile UTIs and negative VCUGs. PIC-VUR was identified in 82% of the patients with febrile UTIs and negative VCUGs. A strong correlation between the ureteral orifice appearance, hydrodistention and the presence of VUR was identified. If the orifice was patulous, it was 38 times more likely to demonstrate VUR. Laterally displaced orifices were 9 times more likely to demonstrate VUR. Also 100% of orifices that hydrodistended were positive for VUR [4].

This multi-institutional registry demonstrated a similar and reproducible incidence of PIC-VUR in patients with recurrent febrile UTIs as Rubenstein's inaugural study. The study also further established a correlation between orifice location and morphology.

## 5. CLINICAL IMPORTANCE OF PIC CYSTOGRAPHY

To further examine whether PIC-VUR is simply a radiographic observation or an entity with clinical relevance, the following studies were performed.

Hagerty and the PIC Cystography Group concluded that PIC-VUR is clinically significant by determining that the incidence rate of febrile UTI is lowered significantly by treatment of VUR identified by PIC. 14 centers enrolled 118 patients with recurrent febrile UTIs, who demonstrated PIC-VUR. Patients were treated with underwent endoscopic injection (104), ureteral reimplantation (3), or antimicrobial prophylaxis (11). Overall, the incidence rate for febrile UTI decreased significantly from 0.16 per case/mo before PIC-VUR treatment to 0.008 per case/mo after treatment. The post treatment rate of febrile UTI in cases treated with antibiotic versus surgery was not significantly different [5].

Noe and Williams also described their experience with PIC cystography and simultaneous dextranomer/hyaluronic acid copolymer injection in 47 children with a history of pyelonephritis and negative VCUG. Success was defined as no further febrile UTIs. Repeated VCUGs were not performed as in the prior studies, as they were negative prior to treatment. A total of 75% of the patients had PIC-VUR and were treated endoscopically with dextranomer/hyaluronic acid copolymer. Three of the patients developed febrile UTIs after surgery and underwent ureteral reimplantation. None of these patients have had recurrent febrile UTIs. Only one patient has had an afebrile UTI during followup. Of the 12 patients who did not have PIC-VUR, each only had 1 febrile UTI, not recurrent UTIs, prior to cystoscopy [5].

Both of these studies further demonstrate that when a patient with febrile UTIs, with no other clear diagnosis, is identified as having PIC-VUR and is treated, they do not have recurrent febrile UTIs. This reinforces the concept that occult reflux identified by PIC cystography in patients with febrile UTIs is clinically significant and that the PIC cystogram is an important testing modality that should be included in the present algorithm of the evaluation of patients with recurrent febrile UTIs.

## 6. THE REPLACEMENT OF POSTOPERATIVE VCUC WITH PIC CYSTOGRAPHY

Pinto et al. researched the feasibility of avoiding the need to perform a VCUG on an awake child after reflux treatment by performing a PIC cystogram immediately after endoscopic injection. Pinto found the PIC cystogram was not useful for this purpose in a study involving 61 patients with VUR identified on VCUG. Patients underwent dextranomer/hyaluronic acid copolymer injection followed by PIC cystography. If the PIC cystogram was positive, no further injection of dextranomer/hyaluronic acid copolymer was given. The results of the PIC cystogram were compared to the VCUG done at 3 months postoperatively. Three ureters had positive PIC cystograms. None of these patients were found to have VUR on postoperative VCUG. Also, 14 patients had persistent VUR on VCUG despite a negative PIC cystogram at the time of injection [6]. In addition, Palmer has also demonstrated no correlation between intraoperative cystography and postoperative conventional cystography [7]. Our anecdotal experience with this method shows similar results.

Currently, there is no evidence to support the use of PIC cystography after endoscopic injection to predict postoperative outcomes. Therefore, it is not recommended to replace a postoperative VCUG with a PIC cystogram at the time of endoscopic correction of VUR.

## 7. PHYSICS OF PIC CYSTOGRAPHY

The impact of intravesical pressure upon the status of PIC-VUR was examined from historical clinical considerations, in vitro simulation study, and clinical examination. Historically, it is commonly held that VUR may be induced in a normal ureteral orifice by conditions which chronically impose supraphysiological pressure such as neuropathic or nonneurogenic neurogenic bladder; however it is commonly held that VUR is not able to be induced by acute application of elevated intravesical pressure [2]. We have demonstrated that when the PIC cystogram is performed as described above the intravesical pressure local to the ureteral orifice pressure is physiological (<20 cm water). In contrast, the practice of hand injection of contrast is associated with a supraphysiological pressure (>100 cm water) [8].

## 8. CLINICAL USE AND FURTHER DIRECTIONS

Currently, there are several widely accepted explanations for recurrent UTIs including the presence of various host and bacterial virulence factors, as well as inadequately treated DES. Nevertheless, it is becoming widely accepted that it is also possible that this type of patient may have occult reflux, not identified on conventional VCUG, that can allow ascent of a lower tract infection to an upper tract infection that is febrile in nature. If so, identification and treatment of this form of occult reflux, PIC-VUR, results in a decrease in recurrent febrile UTIs. The PIC cystogram represents a relatively simple objective way to identify this type of occult VUR that may be clinically significant.

In a recent debate on PIC cystography at the Society of Pediatric Urology it was argued that there is little data evaluating whether or not occult VUR identified by PIC cystography can cause renal injury. In addition, febrile UTIs as described in most of this research on PIC cystography, do not necessarily equate pyelonephritis [9]. While these observations are valid, it is important to note that while it is unknown whether or not occult reflux identified with PIC cystography results in renal scarring, the present evidence clearly demonstrates that treatment of this occult reflux with either prophylactic antibiotics or surgery decreases the rate of recurrent febrile UTIs and its associated morbidity. Even though many of these patients may not have significant renal scarring or be at risk for renal damage, the clinical benefit to these patients is extremely important. However, it will be important to evaluate these important issues as they relate to renal scarring in future studies. In the end, the ability to identify a causative factor that can be treated and reduce or eliminate future febrile infections and the morbidity associated with them is beneficial to both patients and their families.

To more definitively define the clinical significance of PIC cystography, a prospective randomized trial is now underway in which patients who are identified as having PIC-VUR are being randomized into 2 study groups: observation (no antibiotics or surgery) and treatment (endoscopic or open surgical correction of reflux) [10].

## 9. CONCLUSION

Many children with recurrent febrile UTIs do not demonstrate VUR on conventional VCUG. Thus, in such children, there is neither a treatable diagnosis nor an evidence-based treatment plan. This scenario may become associated with significant morbidity such as the need for hospitalization and renal damage. A treatable diagnosis could improve structuring a management strategy. The current research on PIC cystography shows that the PIC cystogram can identify clinically significant occult VUR. When this occult reflux is treated, the incidence of recurrent febrile UTIs is significantly reduced. We conclude that including the performance of PIC cystography in the present algorithm management of patients with recurrent febrile UTIs and normal conventional VCUGs will aid structuring an evidence-based treatment plan. Future prospective randomized studies are currently underway to refine our understanding of the natural history of occult reflux and the role that PIC cystography has in identifying this type of reflux.
